# Multiple Fano-Like MIM Plasmonic Structure Based on Triangular Resonator for Refractive Index Sensing

**DOI:** 10.3390/s18010287

**Published:** 2018-01-19

**Authors:** Nikolina Jankovic, Norbert Cselyuszka

**Affiliations:** BioSense Institute-Research Institute for Information Technologies in Biosystems, University of Novi Sad, Dr Zorana Djindjica 1a, 21000 Novi Sad, Serbia; nikolina@biosense.rs

**Keywords:** Fano resonance, metal-insulator-metal, plasmonic sensor, triangular cavity

## Abstract

In this paper, we present a Fano metal-insulator-metal (MIM) structure based on an isosceles triangular cavity resonator for refractive index sensing applications. Due to the specific feeding scheme and asymmetry introduced in the triangular cavity, the resonator exhibits four sharp Fano-like resonances. The behavior of the structure is analyzed in detail and its sensing capabilities demonstrated through the responses for various refractive indices. The results show that the sensor has very good sensitivity and maximal figure of merit (FOM) value of 3.2 × 10^5^. In comparison to other similar sensors, the proposed one has comparable sensitivity and significantly higher FOM, which clearly demonstrates its high sensing potential.

## 1. Introduction

Surface plasmon polaritons (SPP) represent electromagnetic surface waves occurring at the interface between a dielectric and conductor due to the coupling of electromagnetic fields to collective electron oscillations [1]. Due to the peculiar nature and ability to overcome the diffraction limit and confine light in sub-wavelength dimensions, SPPs have found a number of applications [2,3,4,5]. There have been different SPP guiding structures proposed such as metal nanoparticle chains, metal films, metal-insulator-metal (MIM) slabs, metal grooves, metal strips, and a hybrid Bragg waveguide, among which MIM slabs have attracted significant attention due to their suitability for highly-integrated photonic circuits and a good trade-off between the light confinement and energy loss [6]. Therefore, various devices based on MIM waveguides have been proposed, including switches [7], filters [8,9,10], sensors [11,12], splitters [13], and demultiplexer sensors [14].

When it comes to sensors, their sensitivity and figure of merit (FOM) are critical for overall performance. One of the promising solutions for sensitivity and FOM improvement is the employment of Fano resonances which exhibit an asymmetrical response with very abrupt change between transmittance peak and dip. Although originally discovered in atomic physics [15], Fano resonance has more universal character and it has also been found and used in other fields such as plasmonics, metamaterials, and photonics [16,17]. Generally, Fano resonance occurs due to the interaction between bright (radiative) and dark modes (non-radiative), i.e., between spectrally broad continuum states that can couple to the incident light, and narrow subradiant modes that cannot directly be excited by the light. Such behavior is usually obtained by the introduction of an asymmetry in the structure. However, it has been shown that Fano resonances can be also obtained through mechanisms other than classical near-field interactions and mode hybridization [18].

In MIM plasmonic structures, Fano resonances are usually realized following the classical pattern, i.e., two or three different resonators are employed, one of which effectively creates bright mode, and the other(s) dark mode(s) [19,20,21,22,23,24,25,26,27,28]. There are also structures whose Fano response is based on phase-coupling between two detuned modes of different resonators [29,30,31], interaction between the modes of two identical resonators [32,33], or between different modes of the same resonator [34,35]. In addition, there are designs in which different mechanisms are combined such as those proposed in [36,37], in which interference between different resonant modes of one resonator, the interference between the resonator’s degenerate modes, and the interference between the resonant modes of different resonators, have been employed to realize a triple-Fano response.

As for the geometry of the structures, groove resonators are widely exploited and combined with other resonators such as stub resonators [21,26,28,30], circular [22,25,26] and rectangular cavities [20], as well as with another groove [34]. Other configurations with different types of resonators include those with stub and ring resonators [27,31] and circular cavity and T-shaped waveguide [29]. Moreover, there have been proposed MIM Fano structures with two resonators of the same type such as stub resonators [19], rectangular and square cavities [23,32,36], ring resonators [24], metal strips [33], as well as those that are based on only one resonator-trapezoid cavity [35] and circular cavity [37].

Most proposed sensors have been designed to exhibit single or double Fano resonance, although multiple-Fano responses are preferable since it may provide more reliable results or even multi-sensing applications. However, the proposed multiple-Fano sensors cannot provide good sensitivity, high FOM, and high transmission level simultaneously at all resonances.

In this paper, we present a Fano MIM structure based on an isosceles triangular cavity resonator which exhibits four Fano-like resonances. The feeding lines provide a broad continuum state which is coupled to the four narrow fundamental modes of the resonator. Owing to the feeding scheme, in which the feeding lines are asymmetrically positioned with respect to the triangular resonator, the resonant modes are somewhat disturbed and exhibit asymmetrical Fano-like response rather than classical Lorentzian shape. Moreover, all four resonances exhibit sharp resonances and high transmission magnitudes, and thus the proposed structure has an excellent refractive index sensing potential with good sensitivity and high FOM, overperforming most similar structures.

In the following sections, a detailed analysis of the proposed Fano MIM sensor will be given, together with the eigenmode analysis of the triangular cavity resonator and its behavior within the MIM environment. The sensing capabilities will be demonstrated through the comparison of the responses for different values of the refractive index and the overall performance of the proposed sensor will be discussed.

## 2. Structure Analysis

**Figure 1:**
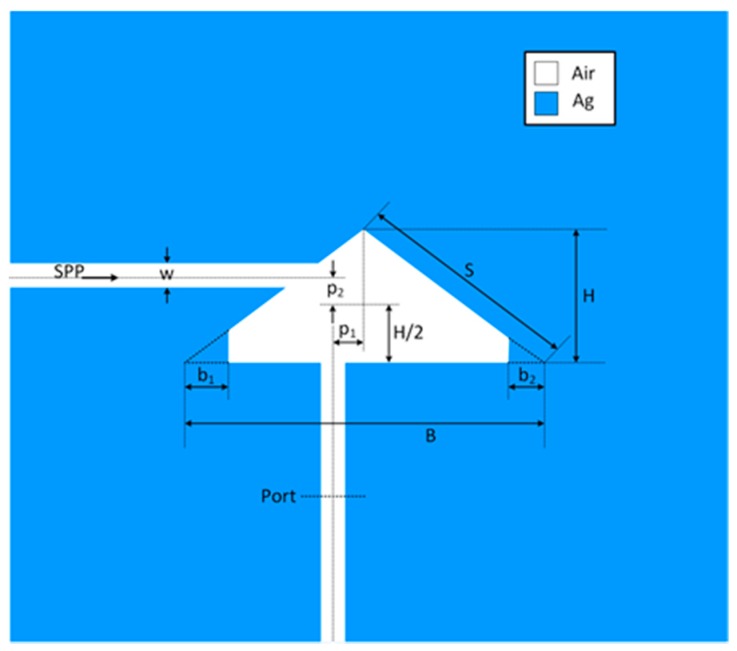
Layout of the proposed Fano MIM sensor with the corresponding geometrical parameters. Two MIM waveguides and triangular resonator are filled with air (marked in white) and surrounded with silver (marked in blue).

The layout of the proposed sensor, together with the corresponding geometrical parameters are shown in Figure 1. It consists of an isosceles triangular cavity resonator with slightly disturbed lower corners, and two MIM waveguides, which are asymmetrically positioned with respect to the cavity. The white area represents air whose refractive index is equal to 1, whilst the blue area represents silver which is characterized using the Drude model [38]

(1) $$\varepsilon_{m}=\varepsilon_{\infty }-\frac{\omega_{p}^{2}}{\omega^{2}+i\omega \gamma },$$

where the permittivity at the infinite frequency *ε*_∞_ is equal to 3.7, plasma frequency *ω_p_* is 1.38 × 10^16^ rad/s, and the damping frequency *γ* equals 2.73 × 10^13^ Hz. The width of the MIM waveguide *w* has been chosen to be 50 nm to provide that only the fundamental transverse magnetic (TM0) mode is supported. The base of the triangular resonator *B* is equal to 600 nm, its height *H* 415 nm, whilst its sides *S* are equal to 513 nm. It should be noted that the dimensions *B*, *H*, and *S* are related to the full triangle as indicated by the dashed lines in Figure 1, and they are fixed throughout the paper. However, the proposed resonator somehow has disturbed corners, i.e., the lower corners are cut and thus additional dimensions have been introduced—*b_1_* and *b_2_*—which indicate the disturbance of the triangular resonator. Also, *p_1_* and *p_2_* represent the distances of the central axes of the MIM waveguides from the symmetry axis and half-height point of the resonator, respectively.

**Figure 2:**
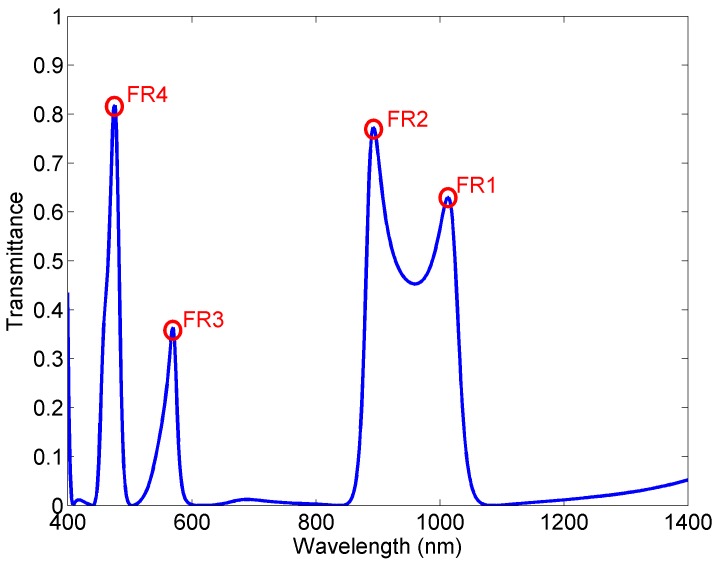
Transmission spectrum of the proposed Fano MIM sensor.

The structure’s response has been investigated using finite element method (FEM) with the Comsol Multiphysics and the transmittance of SPPs is defined as the ratio between the SPP power flows (obtained by integrating the Poynting vector over the waveguide cross section) of the port with and without triangular structure [21,22]. Figure 2 shows the transmission spectrum of the structure with the following dimensions: *b_1_* = 50 nm, *b_2_* = 30 nm, *p_1_* = 30 nm, and *p_2_* = 30 nm, and it reveals that the structure exhibits four narrow asymmetric profiles, i.e., Fano-like resonances at 1012, 892, 570, and 476 nm, marked as FR1, FR2, FR3, and FR4.

The structure can be described using multimode interference coupled mode theory (MICMT) [36,37], i.e., using the expressions

(2) $$\frac{da_{n}}{dt}=\left(j\omega_{n}-\frac{1}{\tau_{n0}}-\frac{1}{\tau_{n1}}-\frac{1}{\tau_{n2}}\right)a_{n}+\kappa_{n1}s_{1+}+\kappa_{n2}s_{2+}$$

(3) $$s_{1-}=-s_{1+}+\underset{n}{\sum }\kappa_{n1}^{*}\gamma_{n}a_{n}e^{j\varphi_{n1}}$$

(4) $$s_{2-}=-s_{2+}+\underset{n}{\sum }\kappa_{n2}^{*}\gamma_{n}a_{n}e^{{j\varphi }_{n2}}$$

where *a_n_*, *ω_n_*_,_ and *τ_n0_* represent the field amplitude, resonant frequency, and the decay time of internal loss of the n-th resonant mode respectively. *τ_n1_* and *τ_n2_* are the decay time of the coupling between the resonator and waveguides, *θ_n1_* and *θ_n2_* are the coupling phases, and *ϕ_1n_* and *ϕ_2n_* are the complex amplitude phases of the n-th resonant mode coupled to the waveguides. *s_i±_* are the field amplitudes in each waveguide (i = 1, 2, for outgoing (−) or incoming (+) from the resonator), whilst *γ_n_* is the normalized coefficient which is approximated as 1.

**Figure 3:**
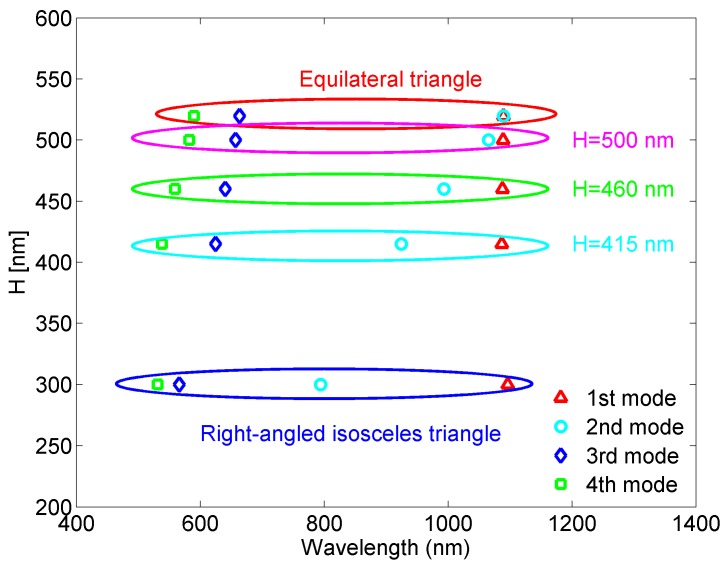
Spectral mode distribution for different types of triangular cavity resonators.

To understand the underlying idea of the proposed sensor and the nature of its response, the behavior of the triangular cavity resonator should be analyzed. Although widely used, triangular resonators have been analytically investigated practically only in the cases of equilateral or right-angled isosceles triangles, primarily due to very complex mode calculus [39,40,41]. Using numerical eigenmode analysis, the resonant wavelengths of the four lowest modes have been calculated for the equilateral triangular resonator with the side length of 600 nm, and the right-angled isosceles triangle with the base equal to 600 nm, Figure 3. One can note that in the case of equilateral triangular resonator two lowest modes have the same spectral position, and the other two are positioned at 664 and 590 nm. On the other hand, the right-angled isosceles triangular resonator exhibits two lowest modes that are relatively distant, and two higher modes that are very closely positioned. Thus, the two triangular cavities are not preferable choices if multiple resonances with even spectral distribution are desired.

To provide more than three resonant modes, which are evenly positioned in the spectrum, we have modified the equilateral triangular resonator in terms of its height, i.e., it has been transformed into isosceles triangle which is not however right-angled. Figure 3 also shows how the resonant wavelengths change with the triangle height and it can be seen that more even modes’ distribution can be obtained in the case of isosceles triangular resonator, which is more appropriate for multiple-sensing applications. For further investigation, we chose the structure whose height is equal to 415 nm.

**Figure 4:**
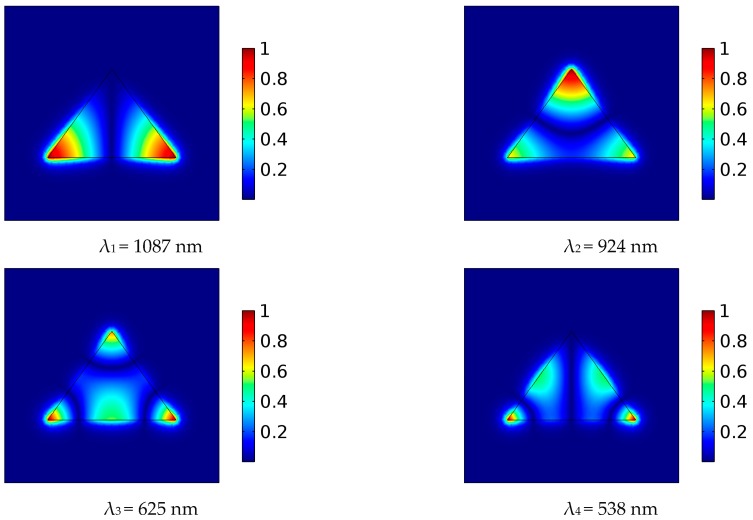
Normalized Hz field distribution at eigenmode resonant frequencies for isosceles triangular resonator.

In order to further understand the nature of the proposed structure, numerical eigenmode analysis of the fully symmetrical isosceles triangular cavity with *H* = 415 nm has been performed and Figure 4 shows normalized H_z_ field distribution of the four lowest fundamental modes.

To achieve Fano-like response at four fundamental modes, we have introduced asymmetry in the structure primarily through the specific position of the MIM waveguides. Namely, feeding positions have not been mirrored with respect to the triangle symmetry axis, but they have been judiciously chosen in a manner that one MIM waveguide is positioned at the point of sufficiently strong field of a mode, and the other at the point of zero or very weak field of the mode. Such an asymmetrical feeding scheme provides that a mode is simultaneously strongly and weakly excited, and consequently transmission peak and transmission dip occur very closely in the spectrum.

Taking into consideration this concept and the field distribution of the four modes, the MIM waveguides have been positioned as shown in Figure 1, since this allows the previously described phenomenon to occur at all four modes. One should note that this would not be possible in case of rectangular or circular cavities due to more even distribution of their modes.

Nevertheless, in the case of fully symmetrical triangular cavity the feeding scheme by itself could not provide very narrow asymmetric profiles at all four resonances, i.e., the phenomenon was not equally profound for all modes. Therefore, additional asymmetry has been introduced by disturbing and cutting the corners of the triangular cavity. We note that the effect of the small disturbances on the field distribution is practically negligible.

**Figure 5:**
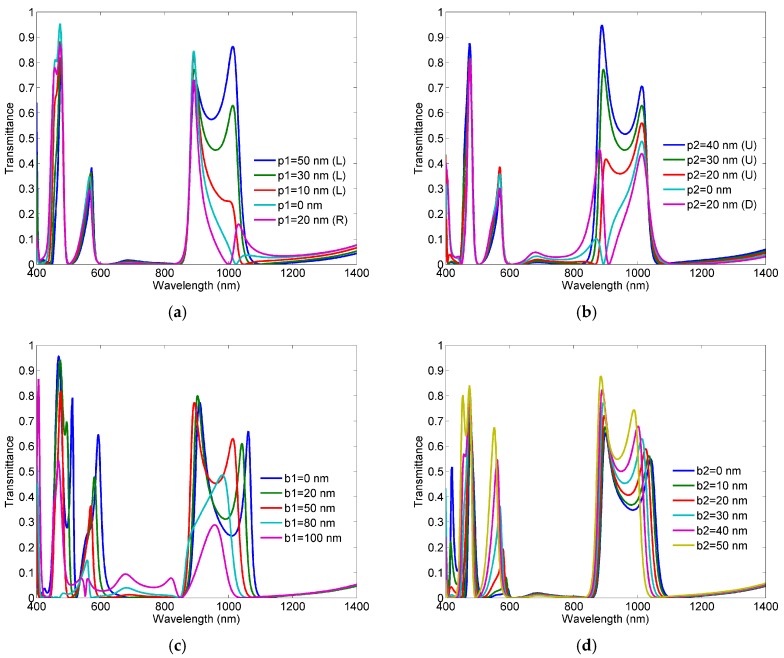
Response of the proposed sensor for different values of the parameter: (**a**) *p_1_*_,_ *L*/*R* denotes left/right position from the symmetry axis; (**b**) *p_2_ U*/*D* denotes upper/down position from the half-height point; (**c**) *b_1_*; (**d**) *b_2_*.

Figure 5 shows responses of the proposed structure for different values of the parameters *p_1_*, *p_2,_ b_1_*, and *b_2_*. For each variation, all other parameters were kept constant.

The variation of the parameter *p_1_* affects to the most extent the first resonant peak FR1, which is expected since, in the considered area of *p_1_* variation, the first mode shows the most abrupt change of the magnetic field. Although one may argue that the structure with *p_1_*= 50 nm exhibits better response in terms of transmittance magnitude, the solution with *p_1_*= 30 nm provides steeper slope at the first two peaks FR1 and FR2, and suppresses the influence of the fifth mode to the fourth one (FR4). By the same token, the change of the parameter *p_2_* influences primarily the second resonance FR2, since it shows the most abrupt change of the magnetic field in the area of the *p_2_* variation.

When it comes to the parameter *b_1_*, its increase provides better slope in the third and fourth Fano-like resonances, FR3 and FR4, however it does so at the expense of the transmission magnitude at the two resonances. Therefore, the value of 50 nm has been chosen as a good trade-off between the steepness of peak-to-dip slope and the transmittance level. Similarly, the variation of the parameter *b_2_* provides steeper peak-to-dip slope in the third and fourth resonances. However, it does so at the expense of the transmittance level and interference of the fourth and fifth modes. Thus, the value of 30 nm represents a good trade-off between these opposite requirements. We note here that both lower corners have been cut and finely tuned since combination of both disturbances has given better results than in the case of only one disturbance.

The previous analysis confirms that multiple Fano-like response with four narrow asymmetric profiles has been obtained primarily through the asymmetric feeding scheme. However, to obtain similar effect for all four modes, additional disturbances had to be introduced in the structure.

## 3. Sensing Potential of the Structure

**Figure 6:**
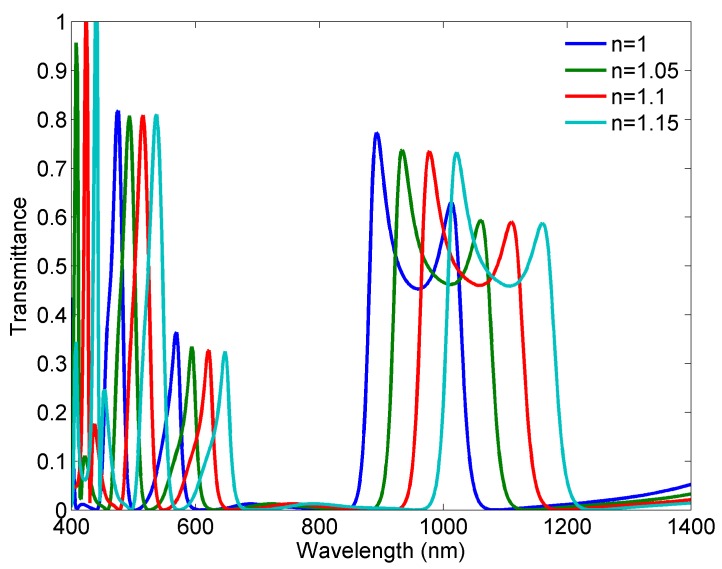
Responses of the sensor for different refractive indices.

Sensing potential of the proposed structure is demonstrated through its response for different refractive indices of the dielectric marked in white in Figure 1. The geometrical parameters of the structure are those given in the previous section. The refractive index has been varied from 1 to 1.15 with the step of 0.05 and the corresponding responses are shown in Figure 6. It can be seen that the Fano-like response is preserved at all four resonances for each response.

The sensitivity of a sensor (nm/RIU) is usually defined as the shift in the resonance wavelength per unit variations of the refractive index [28]. According to the responses, the sensitivities are 986 nm/RIU, 866 nm/RIU, 520 nm/RIU, and 413 nm/RIU at FR1, FR2, FR3, and FR4, respectively. One should not be misled by the relatively low absolute values of the sensitivities at FR3 and FR4, since those sensitivities are related to the wavelength range 400–600 nm, and they actually represent high values relative to the wavelength range. In other words, each resonance is almost equally responsive to the change in the refractive index, if the above-mentioned sensitivities are perceived relatively to the wavelengths of the corresponding resonances.

**Figure 7:**
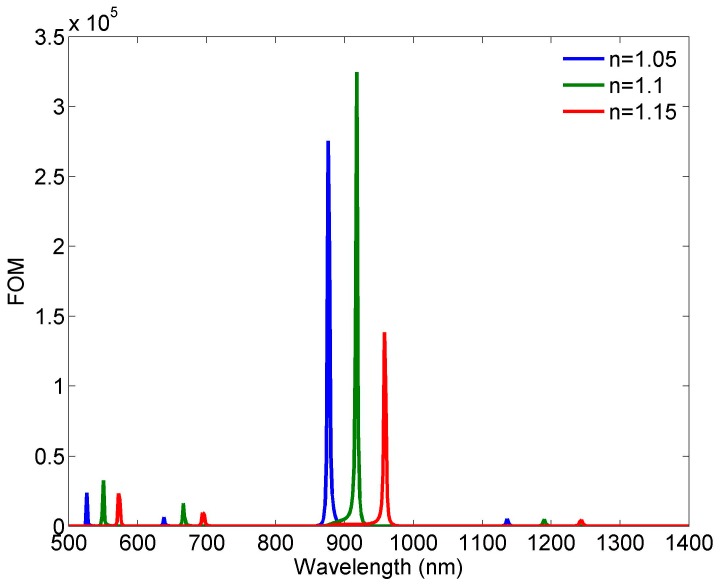
Calculated FOM for different refractive indices.

Another important and more illustrative parameter of a sensor performance is figure of merit (FOM), which is defined as FOM = *ΔT/TΔn* [28]. T denotes the transmittance in the proposed structure and *ΔT/Δn* denotes the transmission change at the fixed wavelength induced by a refractive index change. Figure 7 shows FOMs calculated for the refractive indices 1.05, 1.1, and 1.15 as FOM = (T_n = 1.05/1.1/1.15_ − T_n = 1_)/(T_n = 1_Δn). Although the three FOM curves differ in the maximal values, it can be considered that FOM can reach the values of the order of 10^5^ in the case of FR2, which is significantly greater than the most other proposed sensors. At the same time, the FOMs related to FR1, FR3, and FR4 have the values of 5366/5003/4549, 6465/16410/9762, and 24020/32870/23410, for n = 1.05/1.1/1.15, respectively. In addition, the transmittance levels at all four resonances are sufficiently high, which is not readily achievable.

The proposed sensor is compared to other similar structures in Table 1, where type denotes the number of Fano resonances exhibited by a structure. The proposed sensor has comparable sensitivity, however it considerably outperforms other solutions in terms of the maximal value of FOM and the number of Fano resonances. Namely, whilst most published sensors provide single, dual, or triple Fano resonances, the sensor in this work provides four Fano-like resonances using only one resonator, which allows both for miniaturization and multiple sensing check-points. In comparison to the quad-Fano sensor in [28], the proposed sensor has significantly higher FOMs at all four resonances, whilst the one in [30] has a maximal value one order lower than the proposed sensor. Therefore, the proposed sensor clearly demonstrates high sensing potential.

## 4. Conclusions

In this paper, we proposed a MIM structure based on isosceles triangular resonator for refractive index sensing applications. Due to the specific feeding scheme and asymmetry introduced in the triangular cavity, the sensor exhibits four sharp Fano-like resonances, which allows for excellent sensing performance. A detailed analysis of the sensor has shown that it has very good sensitivity and its maximal value of FOM reaches 3.2 × 10^5^. In comparison to other similar sensors, the proposed one has comparable sensitivity and significantly higher FOM, which clearly demonstrates its high sensing potential.

## Figures and Tables

**Table 1:** Comparison of the proposed sensor and other recently published similar solutions.

Reference	Type	Sensitivity [nm/RIU]	FOM
[20]	dual	888/1187	22,000/29,000
[21]	single	1260	23,000
[22]	single	1277	22,000
[23]	dual	850/1120	74000/170,000
[24]	dual	900/1050	14,400/16,500
[25]	dual	1450/800	35,100/33,500
[26]	triple	1700/2000/1000	7100/8600/7500
[28]	quad	200/600/600/2000	3000/500/1500/200
[29]	single	1114	7961
[30]	quad	700/800/1900/1600	Max 38,000
[31]	single	718	4354
[34]	dual	820/1100	7000/320,000
[36]	triple	600/500/500	3803/816/2947
[37]	triple	850/750/950	100/100/100
This work	quad	416/520/866/986	32,870/16,410/324,600/5003
